# Prevalence and Characteristics of Physicians Engaged in Research in the US

**DOI:** 10.1001/jamanetworkopen.2024.33140

**Published:** 2024-09-24

**Authors:** Alyssa Browne

**Affiliations:** 1 Association of American Medical Colleges, Washington, DC

## Abstract

This cross-sectional study uses the Association of American Medical Colleges 2022 National Sample Survey of Physicians to offer a more complete understanding of the prevalence and characteristics of research-engaged physicians in the US.

## Introduction

There is an incomplete understanding of the prevalence and characteristics of research-engaged physicians in the US.^1^ Most studies focus narrowly on physician-scientists, often defined as National Institutes of Health–funded physicians or physicians for whom research is their primary professional activity,^1,2^ capturing an essential, but incomplete, segment of this population. Using the Association of American Medical Colleges (AAMC) 2022 National Sample Survey of Physicians (NSSP), this study offers a more complete understanding of the prevalence and characteristics of research-engaged physicians.

## Methods

This cross-sectional study uses data from the 2022 NSSP, the second iteration of a national survey of practicing physicians in the US. The 2022 NSSP followed the University of Michigan Population Dynamics and Health Program strategy to recruit a sample of 5917 US-based physicians.^3^ Analytical weights based on American Medical Association data are applied to ensure results are nationally representative based on age group, specialty group, and gender (eTable in Supplement 1).

Respondents self-reported the percentage of their working time spent on research activities in a typical week; respondents who reported any time spent on research were considered research engaged. This study was reviewed by the AAMC institutional review board and received exemption because it does not constitute as human subjects research, as defined in 45 CFR 46. This project is a secondary analysis of deidentified data. This study followed the STROBE reporting guideline.

## Results

Physicians reporting research engagement were demographically reflective of the overall physician workforce. Among the analytical sample of 897 research-engaged physicians (Table 1), only 7.4% (95% CI, 5.0%-10.9%) were part of a racial or ethnic group considered underrepresented in medicine, whereas 62.2% (95% CI, 56.7%-67.4%) were White only, and 28.2% (95% CI, 23.4%-33.6%) were Asian only. Most research-engaged physicians (66.5%; 95% CI, 61.1%-71.4%) were cisgender men, whereas 33.4% (95% CI, 28.4%-38.7%) were cisgender women, and less than 1% identified as another gender (0.01%; 95% CI, 0.00%-0.04%). In this study, 14% (95% CI, 12.6%-15.5%) of all physicians self-reported research engagement (Table 2). However, most research-engaged physicians (83.6%; 95% CI, 79.2%-87.2%) dedicated 10% or less of their weekly hours to research, whereas 12.8% (95% CI, 9.6%-16.8%) spent between 11% and 50% of their time on research. Only 3.6% (95% CI, 2.1%-6.2%) spent more than half of their time on research, representing 0.5% (95% CI, 0.3%-0.9%) of all physicians.

**Table 1.** Sociodemographic Characteristics of Physicians Engaged in Research in the US

Sociodemographic characteristic	No. (%)	
	Research-engaged physicians (n = 897)	All physicians (n = 5917)
Race and ethnicity		
American Indian or Alaska Native	1 (0.0)	11 (0.1)
Asian	244 (28.2)	1416 (25.4)
Black or African American	27 (2.3)	145 (2.2)
Hispanic, Latino, or Spanish origin	28 (3.0)	185 (2.5)
Multiple races	24 (2.2)	123 (2.3)
Native Hawaiian or other Pacific Islander	0	13 (0.2)
Other	22 (1.9)	125 (2.1)
White	524 (62.2)	3744 (64.8)
Not reported	27 (0.0)	155 (0.3)
Aggregated race and ethnicity		
Underrepresented in medicine^a^	80 (7.4)	477 (7.3)
Gender identity		
Male	668 (66.5)	4084 (62.6)
Female	223 (33.4)	1792 (37.1)
Transgender, genderqueer, or other	1 (0.2)	7 (0.2)
Not reported	5 (0.0)	34 (0.1)
Sexual orientation		
Straight or heterosexual	856 (96.4)	5596 (95.1)
Lesbian, gay, bisexual, asexual, or other	32 (2.3)	224 (3.2)
Not reported	9 (1.2)	97 (1.8)
Mean age (95% CI), y	53.2 (52.0-54.4)	53.9 (53.7-54.0)
Disability status		
No disability	838 (94)	5566 (94.4)
Disability	31 (3.7)	195 (2.8)
Do not know	26 (2.1)	146 (2.5)
Not reported	2 (0.2)	10 (0.3)
Citizenship		
Citizen	844 (93.9)	5699 (95.6)
Not a citizen	49 (4.8)	162 (2.6)
Not reported	4 (1.3)	56 (1.8)

All demographic characteristics were self-reported by respondents.

^a^ Includes Hispanic, Latino, or of Spanish origin; American Indian or Alaska Native; Black or African American; or Native Hawaiian or Other Pacific Islander alone or in combination with any other race and ethnicity.

**Table 2.** Professional Characteristics of Physicians Engaged in Research in the US

Professional characteristic	No. (%)	
	Research-engaged physicians (n = 897)	All physicians (n = 5917)
Engaged in research	897 (100.0)	897 (14.0)
Mean time typically worked each week, h	49.1 (47.2-51.1)	45.1 (44.4-45.8)
Time spent on research, %		
0	0	5013 (85.9)
1-10	727 (83.6)	727 (11.7)
11-50	141 (12.8)	141 (1.8)
51-100	29 (3.6)	29 (0.5)
Types of research		
Clinical	726 (83.6)	726 (11.7)
Basic science	73 (6.7)	73 (0.9)
Translational	138 (14.5)	138 (2.0)
Health services	152 (16.7)	152 (2.3)
Community-based	76 (8.1)	76 (1.1)
Educational	190 (20.7)	190 (2.9)
Engaged in multiple types of research	329 (34.5)	329 (4.8)
Degree type		
MD	718 (70.5)	4710 (73)
DO	52 (4.0)	566 (8.1)
Not reported	127 (25.4)	641 (19)
Earned a PhD		
Yes	84 (9.2)	240 (4.2)
No	813 (90.8)	5677 (95.8)
Not reported	0	0
Affiliated with an academic health center		
Yes	588 (61.8)	2203 (36.1)
No	299 (37.6)	3662 (63.0)
Not reported	10 (0.6)	52 (0.9)
Faculty rank (among academically affiliated)		
Not faculty	89 (10.0)	3848 (26.4)
Instructor	49 (4.1)	308 (9.2)
Other	6 (0.8)	97 (2.0)
Assistant professor	171 (25.8)	834 (26.9)
Associate professor	147 (32.2)	550 (22.5)
Professor	123 (27.1)	247 (12.2)
Not reported	3 (0.0)	33 (0.8)
Specialty group		
Primary care	141 (14.6)	1903 (30.9)
Medical specialties	327 (32.9)	1233 (17.5)
Surgery	213 (22.2)	1143 (18.2)
Other	216 (30.4)	1638 (33.4)
Not reported	0	0

Only 9.2% (95% CI, 6.5%-12.8%) of research-engaged physicians had a PhD; 37.6% (95% CI, 32.3%-43.2%) were not academically affiliated, whereas 61.8% (95% CI, 56.2%-67.1%) were. Among academically affiliated research-engaged physicians, 25.8% (95% CI, 19.9%-32.7%) were assistant professors, 32.2% (95% CI, 26.0%-39.2%) associate professors, and 27.1% (95% CI, 21.5%-33.4%) full professors. The rest were instructors, not faculty, or had another role. Research-engaged physicians worked across all specialty types, including one-third in medical specialties (32.9%; 95% CI, 28.5%-37.5%) and 22.2% (95% CI, 18.2%-26.7%) in surgical specialties. The most common type of research reported was clinical (83.6%; 95% CI, 79.0%-87.3%); basic science research was least common (6.7%; 95% CI, 4.5%-10.1%). One-third (34.5%; 95% CI, 29.3%-40.0%) of research-engaged physicians reported engagement in multiple types of research.

## Discussion

The population of research-engaged physicians is characterized by the same racial and ethnic and gender disparities as the overall workforce.^4,5^ Although a limitation of self-reported data is that we cannot confirm that what respondents consider research activities falls within widely accepted definitions of research, these results provide a more complete estimate of the current prevalence of physician research engagement than previously available. Although results suggest a much larger share of physicians are engaged in research than the estimated 1% to 2% who are physician scientists,^1,4^ most research-engaged physicians spend less than 10% of their time on research, and a similarly small proportion (0.5%-1%) of our respondents might be considered physician-scientists based on the percentage of time spent on research. These cross-sectional study findings provide a benchmark estimate for future studies and present evidence that physicians across medical specialties and work settings—including physicians in community-based settings—are actively engaged in a diverse array of research.

## Notes

Supplement 1. **eTable.** Survey Question Text and Response Options

Supplement 2.
Data Sharing Statement
