# Supplementary material for: Prevalence and Characteristics of Physicians Engaged in Research in the US
Source: JAMA Netw Open. 2024 Sep 24;7(9):e2433140. doi: 10.1001/jamanetworkopen.2024.33140 (PMC11423164; doi:10.1001/jamanetworkopen.2024.33140)
Supplement: Supplement 1. — eTable. Survey Question Text and Response Options [file jamanetwopen-e2433140-s001.pdf]

Supplemental Online Content

Browne A. Prevalence and characteristics of physicians engaged in research in the US. *JAMA Netw Open*. 2024;7(9):e2433140.
doi:10.1001/jamanetworkopen.2024.33140

eTable. Survey Question Text and Response Options

This supplemental material has been provided by the authors to give readers additional information about their work.

© 2024 Browne A. *JAMA Network Open.*

eTable. Survey Question Text and Response Options

Variable	Question from which variable was derived	Response options
Research engagement / Time spent on research	During a typical week, approximately what percent of your working time is spent in the following activities?<br><br>Please read all the categories first, before you begin making your best estimates.<br>Please do not include any time you spent on call but not otherwise working.	-Patient care<br>- Combined patient care and teaching or training (e.g., providing patient care while supervising students or residents in clinical settings)<br>-Research<br>-Administration (including institutional service)<br>-Other (please specify)
Types of research	You indicated that you spend time on research. Which of the following types of research do you do? (Please check all that apply.)	-Basic science research<br>-Translational research<br>-Clinical research<br>-Health services or health systems research<br>-Community-based participatory research<br>-Educational research<br>-Other (Please specify)
Race/ethnicity	How do you self-identify?	-Hispanic, Latino/Latina/Latinx, or of Spanish origin (e.g., Argentinean, Colombian, Cuban, Dominican, Mexican, Puerto Rican)<br>-American Indian or Alaskan Native (e.g., Navajo, Blackfeet, Aztec)<br>-Asian (e.g., Chinese, Filipino, Indian, Japanese, Korean, Pakistani)<br>-Black or African American (e.g., African American, Afro-Caribbean)<br>-Native Hawaiian or Other Pacific Islander (e.g., Guamanian, Native Hawaiian, Samoan)<br>-White (e.g., German, Irish, English, Italian)<br>-Other (write-in)

© 2024 Browne A. *JAMA Network Open.*

Gender identity	What best describes your current gender identity? (Check all that apply)	-Man<br>-Woman<br>-Trans man<br>-Trans woman<br>-Genderqueer/Gender non-conforming<br>-Non-binary<br>-Other (please specify)
Sexual orientation	What best describes your current sexual orientation?	-Bisexual<br>-Gay or Lesbian<br>-Heterosexual or straight<br>-Asexual<br>-Pansexual<br>-Other (please specify)
Disability status	The Americans with Disabilities Act (ADA) defines a person with a disability as a person who has a physical or mental impairment that substantially limits one or more major life activities. Do you have a disability as defined by the ADA?	-Yes<br>-No<br>-I don't know
Citizenship	What is your US citizenship status?	-U. S. citizen:, U.S.-born or born abroad to a U.S. citizen<br>-U.S. citizen:, Naturalized<br>-Not a citizen, Legal Permanent Resident<br>-Not a citizen, nor a Legal Permanent Resident
Mean hours typically worked	During your last typical week of work (excluding any week with leave), approximately how many hours were you working?<br><br>(Please include time working away from your usual place of work, such as patient related documentation or consultations with other physicians performed at home or in other locations. Please do not include any hours you spent on call but not otherwise working. We will ask you about time spent on call in a separate question.)	Open-ended, allowable range: 0-168 hours
Degree type	Was your medical school MD-granting or DO-granting?	-MD-granting<br>-DO-granting
Earned a PhD	What other graduate or professional degrees do you hold, if any? (Please select all that apply.)	-No other graduate or professional degrees<br>-PhD<br>-MPH

© 2024 Browne A. *JAMA Network Open.*

		-DrPH<br>-MBA<br>-Other (please specify)
Affiliated with an Academic Health Center (AHC)	Are you currently affiliated (e.g., admitting or consulting privileges) with an Academic Health Center (also called an Academic Medical Center) or teaching hospital (including paid or volunteer, full-time or part-time)?	-Yes<br>-No
Faculty rank	Which of the following best describes your current faculty appointment position with a medical school? (If more than one type of faculty appointment, please select the one in which you spend the most time.)	-Professor<br>-Associate Professor<br>-Assistant Professor<br>-Instructor<br>-Other (please specify)

Note: Age and 4-category specialty group were merged in from the AMA Physician Masterfile. The 4-category specialty group variable included the following categories: medical specialties, primary care, surgery, and other.

© 2024 Browne A. *JAMA Network Open.*
