# Supplementary material for: Prevalence and Characteristics of Physicians Engaged in Research in the US
Source: JAMA Netw Open. 2024 Sep 24;7(9):e2433140. doi: 10.1001/jamanetworkopen.2024.33140 (PMC11423164; doi:10.1001/jamanetworkopen.2024.33140)
Supplement: Supplement 2. — Data Sharing Statement [file jamanetwopen-e2433140-s002.pdf]

Data Sharing Statement

Browne. Prevalence and Characteristics of Physicians Engaged in Research in the US. *JAMA Netw Open*. Published September 24, 2024. doi:10.1001/jamanetworkopen.2024.33140

Data
Data available: No
